# MIG-10 Functions with ABI-1 to Mediate the UNC-6 and SLT-1 Axon Guidance Signaling Pathways

**DOI:** 10.1371/journal.pgen.1003054

**Published:** 2012-11-29

**Authors:** Yan Xu, Christopher C. Quinn

**Affiliations:** Department of Biological Sciences, University of Wisconsin–Milwaukee, Milwaukee, Wisconsin, United States of America; University of California San Diego, United States of America

## Abstract

Extracellular guidance cues steer axons towards their targets by eliciting morphological changes in the growth cone. A key part of this process is the asymmetric recruitment of the cytoplasmic scaffolding protein MIG-10 (lamellipodin). MIG-10 is thought to asymmetrically promote outgrowth by inducing actin polymerization. However, the mechanism that links MIG-10 to actin polymerization is not known. We have identified the actin regulatory protein ABI-1 as a partner for MIG-10 that can mediate its outgrowth-promoting activity. The SH3 domain of ABI-1 binds to MIG-10, and loss of function of either of these proteins causes similar axon guidance defects. Like MIG-10, ABI-1 functions in both the attractive UNC-6 (netrin) pathway and the repulsive SLT-1 (slit) pathway. Dosage sensitive genetic interactions indicate that MIG-10 functions with ABI-1 and WVE-1 to mediate axon guidance. Epistasis analysis reveals that ABI-1 and WVE-1 function downstream of MIG-10 to mediate its outgrowth-promoting activity. Moreover, experiments with cultured mammalian cells suggest that the interaction between MIG-10 and ABI-1 mediates a conserved mechanism that promotes formation of lamellipodia. Together, these observations suggest that MIG-10 interacts with ABI-1 and WVE-1 to mediate the UNC-6 and SLT-1 guidance pathways.

## Author Summary

To form neural circuits, axons must navigate through the developing nervous system to reach their correct targets. Axon navigation is led by the growth cone, a structure at the tip of the growing axon that responds to extracellular guidance cues. Many of these guidance cues and their receptors have been identified. However, much less is known about the internal signaling events that give rise to the structural changes required for growth cone steering. A key component of the internal response is MIG-10, a protein that becomes asymmetrically localized in response to the extracellular cues. MIG-10 is thought to serve as a scaffold that can spatially control outgrowth-promoting proteins within the growth cone. However, we do not know the identity of the outgrowth-promoting proteins that associate with MIG-10. Here we report that MIG-10 associates physically with the actin regulatory protein ABI-1. We present genetic evidence indicating that ABI-1 functions downstream of MIG-10 to mediate its outgrowth-promoting activity. Additional genetic evidence indicates that these proteins function in both attractive and repulsive guidance signaling pathways. We also present evidence suggesting that the connection between MIG-10 and ABI-1 represents a phylogenetically conserved mechanism for the control of cellular outgrowth.

## Introduction

Axons navigate to their targets in the developing nervous system by making a series of responses to extracellular guidance cues [1]–[6]. Several conserved families of guidance cues have been identified, including the netrins and slits. These guidance cues activate receptors on the growth cone at the tip of the growing axon, causing a directional response that steers the axon either towards or away from the source of guidance cue. A key component of the mechanism that drives the directional response to guidance cues is the asymmetric accumulation of f-actin within the growth cone. For instance, *in vitro* growth cone turning assays have shown that actin is asymmetrically polymerized to the side of the growth cone closest to a source of netrin, which is thought to cause the growth cone to turn towards the source of netrin [7]. Likewise, asymmetric actin distribution has also been observed in growth cones migrating *in vivo* [8], [9].

Although many actin regulatory proteins have been implicated in the control of growth cone morphology, we do not understand how these proteins are coordinated to cause a directional response to guidance cues [10]. For example, the WVE-1 (Wave) complex activates the ARP2/3 actin-nucleating complex to control the formation of growth cone filopodia [11], [12]. Furthermore, loss of function of WVE-1 or ARP2/3 components causes defects in axon guidance [11], [13], [14]. However, we do not know how the activity of this complex is controlled to give rise to the asymmetry that underlies growth cone guidance. In particular, it is difficult to understand how shallow gradients of guidance cues could be transformed into the sharply polarized outgrowth-promoting activity that is required for a directional response.

MIG-10 may provide a key to understanding how guidance signals are transformed into sharply localized actin-based outgrowth activity. MIG-10 is a cytoplasmic outgrowth-promoting protein that becomes sharply localized in response to the UNC-6 guidance cue [15]–[19]. The role of MIG-10 in guidance has been studied in the HSN neuron of *C. elegans*, which extends an axon ventrally, towards a source of UNC-6 guidance cue. In response to UNC-6, the UNC-40 (DCC) receptor becomes asymmetrically localized to the side of the cell closest to the source of UNC-6. This in turn, leads to the asymmetric localization of MIG-10 to the side of the cell closest to the source of UNC-6. MIG-10 has an outgrowth-promoting activity, thereby causing axon growth towards the source of UNC-6. However, the mechanisms that mediate the outgrowth-promoting activity of MIG-10 are not understood.

Although MIG-10 and its homolog lamellipodin are thought to play a major role in inducing actin polymerization, the mechanisms that mediate this effect are not known [20]. Knockdown of lamellipodin in fibroblasts results in a severe reduction in polymerized f-actin, with large areas devoid of the normal meshwork of f-actin. The Ena/VASP actin regulatory proteins can physically interact with lamellipodin. However, loss of all Ena/VASP function in fibroblasts does not produce the severe defects in actin polymerization that occur with knockdown of lamellipodin. Likewise, in *C. elegans* axon guidance, complete loss of Ena/VASP (UNC-34) function results in axon guidance defects that are far weaker than those observed after complete loss of MIG-10 function [18]. Furthermore, complete loss of UNC-34 function does not reduce the outgrowth-promoting activity of MIG-10 [16]. Together, these observations indicate that the actin-polymerizing activity of lamellipodin and MIG-10 must be explained primarily through interaction with an effector other than UNC-34 (Ena/VASP).

Here, we present evidence that the outgrowth-promoting activity of MIG-10 is mediated through interactions with ABI-1 and WVE-1. Furthermore, our genetic data indicate that MIG-10 functions with both ABI-1 and WVE-1 to mediate the UNC-6 and SLT-1 guidance signaling pathways. ABI-1 and WVE-1 are part of a well-characterized complex that promotes actin polymerization by activating the actin-nucleating activity of the ARP2/3 complex [21]–[26]. Thus, our observations suggest a model where MIG-10 interacts with ABI-1 and WVE-1 to direct actin polymerization in response to guidance cues.

## Results

### Identification of ABI-1 as a potential component of the MIG-10 scaffold

**Figure 1:**
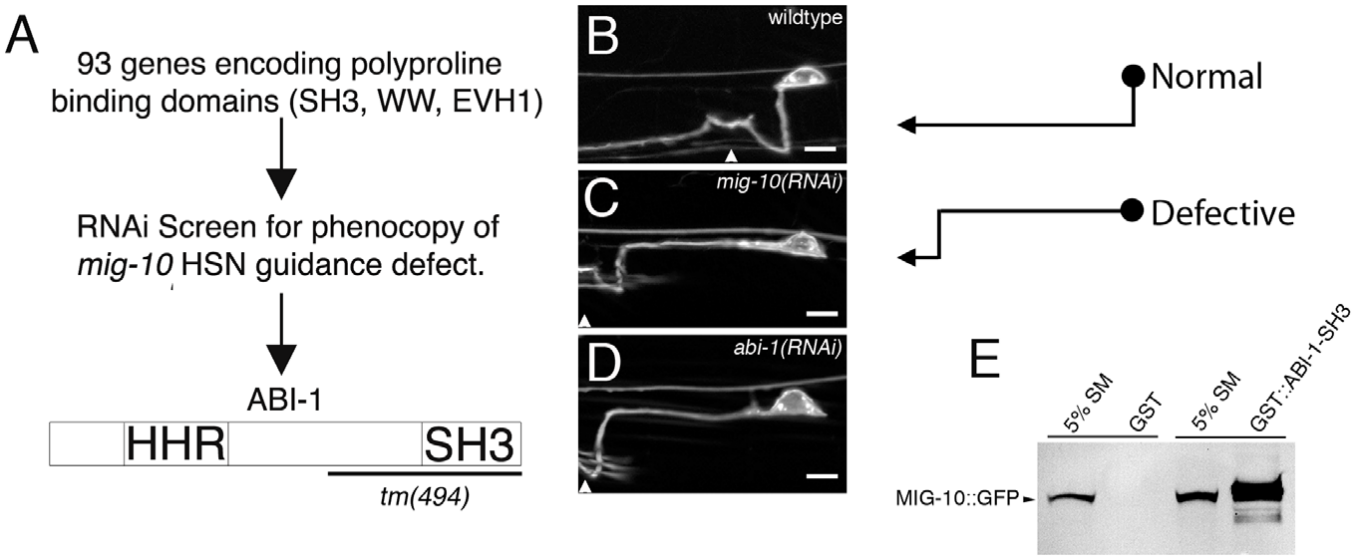
MIG-10 interacts physically with ABI-1. (A) Diagram of the RNAi screening strategy used to identify ABI-1. A sublibrary of RNAi clones encoding proline-binding domains (SH3, WW, EVH1) was created. Each clone was screened for the ability to phenocopy the HSN ventral guidance defect observed in *mig-10* loss of function mutants. This strategy led to the identification of ABI-1 as a potential interaction partner for MIG-10. In this study we have utilized the *abi-1(tm494)* allele, which is predicted to truncate the ABI-1 protein as indicated by the bracket. (B) Example of HSN axon in wild-type animals. The axon makes a direct ventral migration. (C) Example of HSN ventral guidance defect observed in *mig-10(RNAi)* animals. The axon migrates laterally prior to turning ventrally. (D) Example of HSN ventral guidance defect observed in *abi-1(RNAi)* animals. The HSN axon was observed with an *unc-86::myrGFP* transgene. Arrowheads mark approximate position of the vulva. Note that the migration of the HSN cell body was also affected by *mig-10(RNAi)* and *abi-1(RNAi)*. However, previous analysis has indicated that HSN axon guidance defects are not secondary consequences of defects in cell body migration [18], [27]. Scale bars represent 5 µm. (E) MIG-10 binds to the SH3 domain of ABI-1. MIG-10::GFP was incubated with the SH3 domain of ABI-1 fused to GST (GST::ABI-1-SH3) or GST as a control. Bound material was detected by western blotting with an antibody to GFP. For reference, an amount equivalent to 5% of the MIG-10::GFP starting material was run on a gel (5% SM).

MIG-10 is hypothesized to serve as a scaffold that can asymmetrically localize outgrowth-promoting proteins. However, the identities of these outgrowth-promoting proteins are unknown. We hypothesized that these outgrowth-promoting proteins are likely to bind to the polyproline motifs in MIG-10. Therefore, we searched for proteins that include polyproline-binding domains (SH3, WW, and EVH1 domains) for potential function in the MIG-10 pathway. To do this, we constructed an RNAi sublibrary for genes that encode polyproline-binding domains and screened for RNAi clones that phenocopy loss of *mig-10* function in the HSN neuron (Figure 1). The HSN axon normally migrates ventrally to the ventral nerve cord (Figure 1B). In *mig-10* null mutants, 44±4.4% of the HSN axons migrate anteriorly before turning ventrally [18]. We observed this same phenotype in *mig-10(RNAi)* animals, but with a penetrance of 21.6±4.6% (see Figure 1C). From our screen, we identified *abi-1(RNAi)*, which had a penetrance of 15.1±3.6% HSN guidance defects (see Figure 1D). The migration of the HSN cell body was also affected by *mig-10(RNAi)* and *abi-1(RNAi)*. However, previous analysis has indicated that HSN axon guidance defects are not secondary consequences of defects in cell body migration [18], [27].

Since ABI-1 has an SH3 domain, and MIG-10 has consensus-binding sites for SH3 domains, we tested for a physical interaction between the SH3 domain of ABI-1 and MIG-10 (Figure 1E). We found that MIG-10 binds to the SH3 domain of ABI-1 fused to GST (GST::ABI-1-SH3). By contrast, MIG-10 did not bind to GST alone. Two concurrent studies have also found that MIG-10 can bind to full length ABI-1, using yeast 2 hybrid and also co-immunoprecipitation [28], [29]. Together, these observations identify ABI-1 as a potential member of the MIG-10 outgrowth-promoting complex.

### ABI-1 functions in UNC-6 and SLT-1 signaling

The AVM and PVM neurons are ideal for studying ventral guidance because their axons are guided ventrally by both attraction towards a source of UNC-6 guidance cue and by repulsion from a source of the SLT-1 guidance cue. Previous work with these neurons has indicated that MIG-10 functions in both the UNC-6 and SLT-1 signaling pathways [16], [17]. In these experiments, null alleles were used to remove function of either the UNC-6 or SLT-1 guidance cues. Since *unc-6; slt-1* double null mutants exhibit guidance defects that are nearly fully penetrant, UNC-6 and SLT-1 are thought to be the predominant guidance cues responsible for AVM and PVM axon guidance [16], [17], [30], [31]. Therefore, the function of either guidance cue can be assayed by removing the function of the other cue. For example, a null mutation in *mig-10* can enhance guidance defects in the *unc-6* null mutant background, indicating that *mig-10* functions in SLT-1 signaling. Likewise, a null mutation in *mig-10* can also enhance defects in the *slt-1* null mutant background, indicating that *mig-10* functions in UNC-6 signaling. To determine if ABI-1 also functions in UNC-6 and SLT-1 signaling, we repeated these experiments with an *abi-1* loss of function mutation.

**Figure 2:**
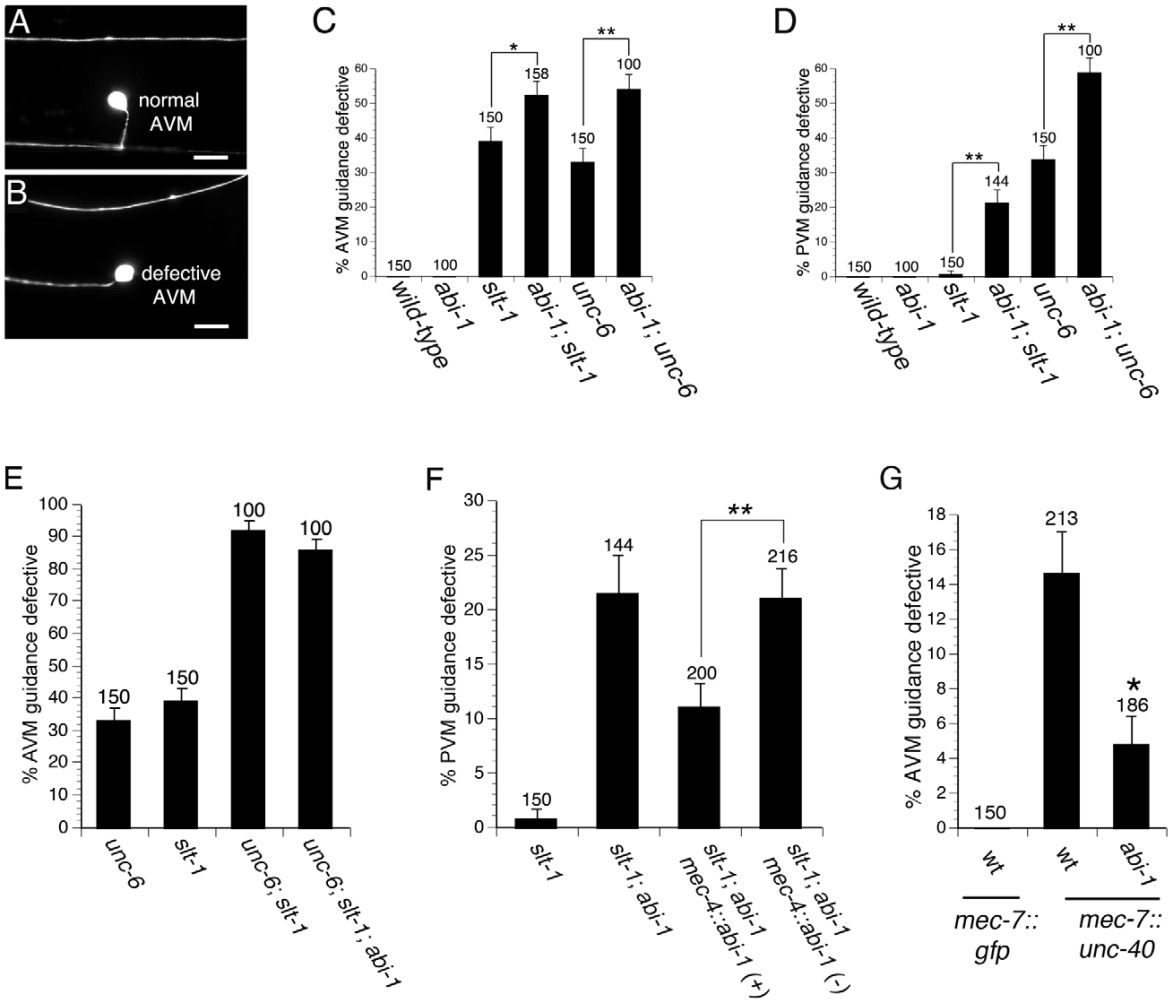
ABI-1 is involved in both UNC-6 and SLT-1 signaling pathways. (A) Example of AVM neuron with normal ventral guidance. (B) Example of AVM neuron with defective ventral guidance. (C–D) Genetic interactions between *unc-6* and *abi-1* as well as between *slt-1* and *abi-1* in the AVM (C) and PVM (D), indicate that ABI-1 functions in both the UNC-6 and SLT-1 signaling pathways. (E) Loss of *abi-1* function does not enhance defects in the *unc-6; slt-1* mutant background. (F) ABI-1 functions cell autonomously to mediate axon guidance. A *mec-4::abi-1* transgene suppresses PVM ventral axon guidance defects in *slt-1; abi-1* double mutants. *mec-4::abi-1(−)* represents animals that have lost the *mec-4::abi-1* transgene during mitosis. These animals serve as controls and were scored simultaneously on the same slides as the *mec-4::abi-1(+)* animals, which carry the transgene. Data was combined from 2 independently derived transgenic lines, *cueEx1* and *cueEx2*, which showed similar results. (G) The *abi-1(tm494)* loss of function mutation suppresses guidance defects in AVM neurons overexpressing UNC-40. Scale bars are 5 µM. Error bars represent standard error of the proportion. Brackets indicate statistically significant difference, Z test for proportions (*p<0.05, **p<0.005). The AVM axon was visualized with the *zdIs5* transgene (*mec-4::gfp*). Scale bars are 10 µM. Alleles used were *unc-6(ev400)* null, *slt-1(eh15)* null, and *abi-1(tm494)* loss of function.

To determine if ABI-1 functions in the UNC-6 signaling pathway, we examined *abi-1(tm494); slt-1(eh15)* double mutants. The *abi-1(tm494)* mutation is a hypomorphic loss of function allele [32], whereas the *slt-1(eh15)* is a null allele. In this *slt-1* null mutant background, the AVM and PVM axons are guided by attraction towards UNC-6. Thus, any enhancement of guidance defects in the *abi-1; slt-1* double mutant would indicate that ABI-1 functions in the UNC-6 pathway. Indeed, we found that in *abi-1; slt-1* double mutants, both AVM and PVM ventral guidance errors were enhanced relative to *slt-1* single mutants, indicating that ABI-1 is involved in the UNC-6 attractive signaling pathway (Figure 2A–2D).

To determine if ABI-1 functions in the SLT-1 signaling pathway, we examined *abi-1(tm494); unc-6(ev400)* double mutants. In this *unc-6* null mutant background, these axons are guided by repulsion from SLT-1. Both AVM and PVM axon guidance errors were significantly enhanced by loss of *abi-1* function in *abi-1; unc-6* double mutants, indicating that ABI-1 is involved in the SLT-1 repulsive signaling pathway (Figure 2A–2D). Despite being involved in both UNC-6 and SLT-1 signaling, we did not observe any AVM or PVM guidance defects in *abi-1* single mutants, suggesting that ABI-1, like MIG-10, functions redundantly with other proteins to mediate UNC-6 and SLT-1 signaling.

Although double mutant analysis suggests that the AVM and PVM axons are guided predominately by UNC-6 and SLT-1, we can not exclude the possibility of a third guidance pathway. To determine if ABI-1 might function in a third pathway, we constructed an *unc-6; slt-1; abi-1* triple mutant. Neither AVM nor PVM axon guidance defects were enhanced in the *unc-6; slt-1; abi-1* triple mutant relative to the *unc-6; slt-1* double mutant (Figure 2E and Figure S1). These observations do not support a role for ABI-1 in a third guidance pathway.

To determine if ABI-1 functions cell autonomously to mediate the axon guidance, we conducted a transgenic rescue experiment in *abi-1; slt-1* double mutants (Figure 2F). The *mec-4* promoter was used to drive expression of ABI-1 in the six touch neurons, including the PVM neuron. Transgenic expression of ABI-1 in the PVM neuron partially rescued the ventral guidance defects in the *abi-1; slt-1* double mutants. By contrast, siblings that had lost the transgenic array did not show rescue of the ventral guidance defects. These observations indicate that ABI-1 functions cell autonomously to mediate the UNC-6 signaling pathway.

Since we found that ABI-1 functions in the UNC-6 signaling pathway, we also wanted to determine if ABI-1 functions downstream of UNC-40, the receptor for UNC-6. To determine if ABI-1 functions downstream of UNC-40, we used a *mec-7::unc-40* transgene to create an UNC-40 gain of function phenotype. The *mec-7::unc-40* transgene caused ventral axon guidance defects in the AVM and PVM neurons (Figure 2G and Figure S2). These guidance defects are suppressed by loss of *abi-1* function in both the AVM and PVM neurons. These observations suggest that ABI-1 functions downstream of UNC-40.

### MIG-10 functions with ABI-1 and WVE-1 to mediate ventral guidance of the HSN axon

**Figure 3:**
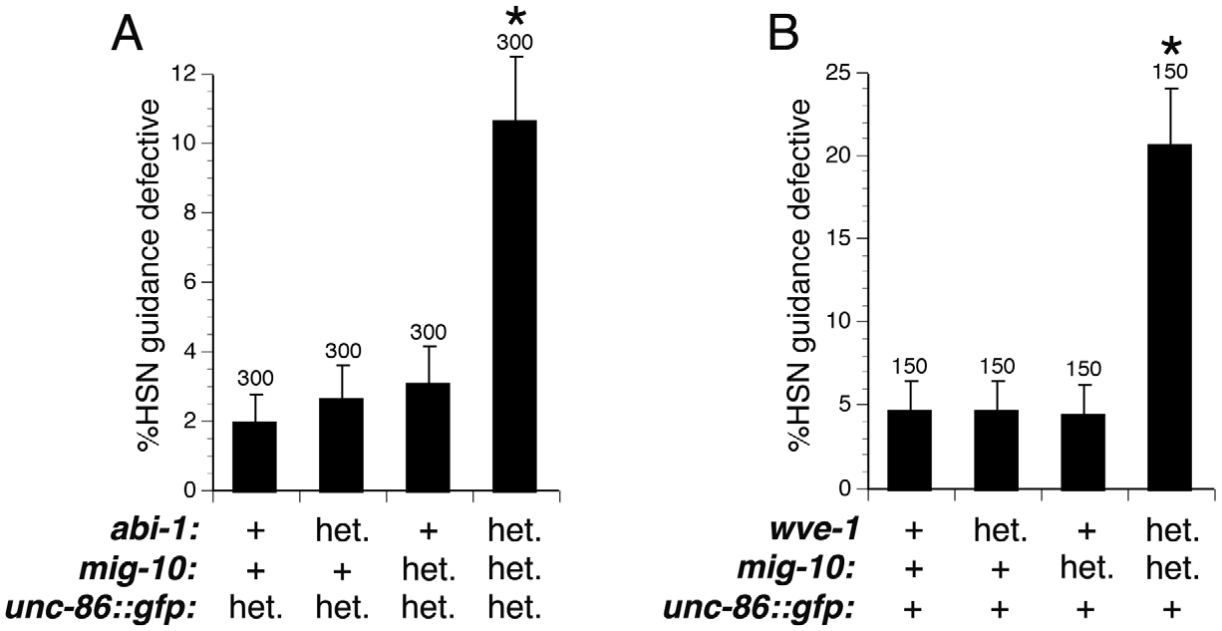
Dosage-sensitive genetic interactions indicate that MIG-10, ABI-1, and WVE-1 function together to mediate axon guidance. (A) Transheterozygous genetic interaction between *abi-1(tm494)* and *mig-10(ct41)*. HSN ventral guidance in animals heterozygous for either *abi-1(tm494)* or *mig-10(ct41)* was comparable to those in wild-type animals. Animals transheterozygous for *abi-1(tm494)* and *mig-10(ct41)* had significantly greater ventral guidance errors compared to wild-type animals. Heterozygous animals were constructed by crossing *unc-86::myrgfp* males with *abi-1(tm494)* or *mig-10(ct41)* hermaphrodites and scoring F1 cross progeny. Transheterozygous animals were constructed by crossing *abi-1(tm494)*; *unc-86::myrgfp* males with *mig-10(ct41)* hermaphrodites and scoring F1 cross progeny. (B) Transheterozygous genetic interaction between *wve-1(ok3308)* and *mig-10(ct41)*. HSN ventral guidance was comparable to wild-type in animals heterozygous for *wve-1(ok3308)* or *mig-10(ct41).* HSN ventral guidance in animals heterozygous for either *wve-1(ok3308)* or *mig-10(ct41)* was comparable to those in wild-type animals. Animals transheterozygous for *wve-1(ok3308)* and *mig-10(ct41)* had significantly greater ventral guidance errors compared to wild-type animals. Similar results were obtained using the *wve-1(ne350)* allele instead of the *wve-1(ok3308)* allele. The hT2 balancer covers both *wve-1* and *mig-10* and was used to construct *wve-1* heterozygotes, *mig-10* heterozygotes, and the *wve-1; mig-10* transheterozygotes. The *kyIs262* transgene (*unc-86::myrgfp*) was used for observing the HSN axon. For the labels in both (A) and (B), “het.” means heterozygous for the mutant or for the *unc-86::myrgfp* transgene. Whereas, “+” means homozygous for the wild-type gene or homozygous for the *unc-86::myrgfp* transgene. Brackets indicate statistically significant difference between transheterozygotes and single heterozygotes, Z test for proportions (*p<0.0005).

The physical association between MIG-10 and ABI-1 suggests that they could function together to regulate axon guidance. To study genetic interactions between *mig-10* and *abi-1*, we used the HSN ventral axon migration, since single mutants in *abi-1* or *mig-10* produce guidance defects in this neuron. Homozygous *mig-10(ct41)* null mutants have guidance defects with a penetrance of 44±4.4% [18]. Homozygous *abi-1(tm494)* hypomorphic loss of function mutants have HSN guidance defects with a penetrance of 14±3.4% (n = 100). To ask if ABI-1 and MIG-10 function together, we used dosage sensitive genetic analysis (Figure 3A). Both the *abi-1* and *mig-10* mutations were recessive, as neither *mig-10* heterozygotes (*mig-10/+*) nor *abi-1* heterozygotes (*abi-1/+*) had guidance errors in excess of wild-type animals. To test for a genetic interaction between *mig-10* and *abi-1*, we examined HSN axon guidance in animals transheterozygous for mutations in *mig-10* and *abi-1*, that is containing one mutant and one wild-type copy of each of these genes (*mig-10/+; abi-1/+*). These transheterozygous mutants had HSN guidance defects with a penetrance of 10.7±1.8% (Figure 3A). Consistent with the physical association between MIG-10 and ABI-1, these observations indicate that ABI-1 functions with MIG-10 to regulate axon guidance.

Since *mig-10* interacts genetically with *abi-1*, we also wanted to test for interaction between *mig-10* and *wve-1*. Studies of the mammalian homologs of ABI-1 and WVE-1, known as Abi1 and Wave, have indicated that Abi1 binds to Wave to enhance its ability to promote lamellipodial protrusion by activating the ARP2/3 complex to promote nucleation and branching of f-actin [21], [22], [24], [25]. Likewise, genetic studies in *C. elegans* have indicated that ABI-1 and WVE-1 are required for subcellular enrichment of f-actin and for the formation of cellular protrusions during cell migration [23]. To determine if WVE-1 is involved in HSN ventral guidance, we examined homozygous *wve-1(ok3308)* mutants that had been maternally rescued. We found that these *wve-1* mutants had HSN guidance defects with a penetrance of 13±3.3% (n = 150), suggesting that WVE-1 is involved in HSN ventral guidance. To determine if MIG-10 functions with WVE-1, we tested for dosage-sensitive genetic interaction between *mig-10* and *wve-1* (Figure 3B). Both *wve-1(ok3308)* and *mig-10(ct41)* are recessive, as neither *mig-10* heterozygotes (*mig-10/+*) nor *wve-1* heterozygotes (*wve-1/+*) had guidance errors in excess of wild-type animals. Animals transheterozygous for *mig-10(ct41)* and *wve-1(ok3308), (mig-10)/+; wve-1/+)*, had HSN guidance errors with a penetrance of 20.7±3.3% (Figure 3B). We repeated this experiment with the *wve-1(ne350)* allele [23] and found that animals transheterozygous for *mig-10(ct41)* and *wve-1(ne350)* had HSN guidance errors with a penetrance of 18±2.7% (n = 200). Together, these results indicate that MIG-10 functions with ABI-1 to regulate guidance.

### ABI-1 and WVE-1 mediate the outgrowth-promoting activity downstream of MIG-10

Previous work has indicated that MIG-10 has an outgrowth-promoting activity and that the actin regulatory protein UNC-34 can interact with MIG-10 [16], [17]. However, complete loss of UNC-34 function does not reduce the outgrowth-promoting activity of MIG-10, indicating that UNC-34 does not account for MIG-10's outgrowth promoting activity.

**Figure 4:**
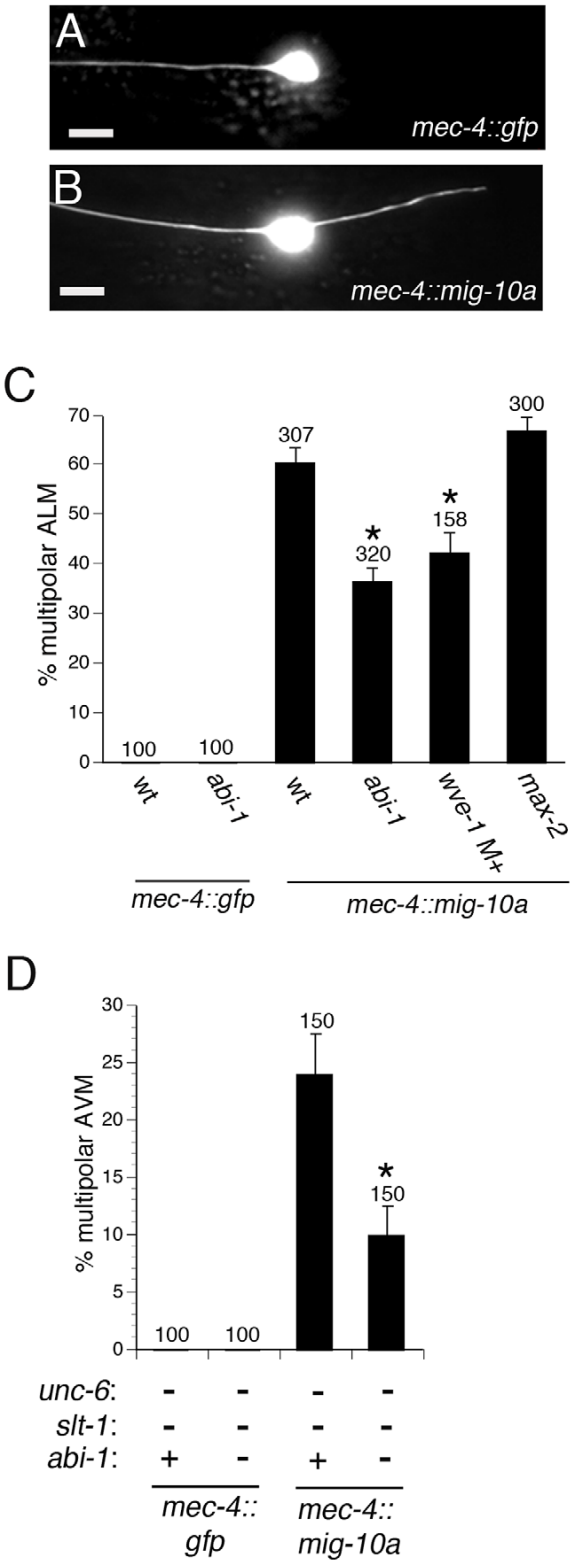
ABI-1 and WVE-1 mediate outgrowth-promoting activity downstream of MIG-10. (A) Example of normal ALM neuron with a single anterior axon. (B) Example of ALM multipolar defect caused by transgenic expression of MIG-10A by the *mec-4::mig-10a* transgene. (C) Loss of function mutations *abi-1(tm494)* and *wve-1(ok3308)* suppress MIG-10 transgenic expression phenotype. The *max-2(nv162)* mutation, a likely null, does not suppress the MIG-10 transgenic expression phenotype. The *wve-1(ok3308)* mutants were maternally rescued. The AVM axon was visualized with a *zdIs5* transgene (*mec-4::gfp*). (D) The *abi-1(tm494)* loss of function mutation suppresses the AVM multipolar phenotype that results from transgenic expression of MIG-10 in the *unc-6; slt-1* double null mutant background. *Statistically significant difference compared to wild-type or *unc-6; slt-1* double mutant, z-test for proportions (p<0.005).

Since ABI-1 and WVE-1 are part of a complex that can promote lamellipodial protrusion, we asked if ABI-1 and WVE-1 can mediate the outgrowth-promoting activity of MIG-10. To test this hypothesis, we determined if loss of WVE-1 function or loss of ABI-1 function could suppress the outgrowth-promoting activity of MIG-10. The ALM neuron normally has a single axon that grows towards the anterior (Figure 4A). Transgenic expression of MIG-10 in the ALM neuron causes the growth of a second posterior process (Figure 4B). The growth of this second process is suppressed by loss of function mutations in *abi-1* or *wve-1* (Figure 4C). By contrast, *max-2(nv162)*, a likely null mutation, had no effect on the growth of the second process. The lack of an effect of the *max-2* mutation is expected because MAX-2 is thought to regulate axon guidance by functioning in a pathway that is parallel to MIG-10 [18]. Together, these observations indicate that ABI-1 and WVE-1 function downstream of MIG-10 to mediate its outgrowth-promoting activity.

We also examined a role for ABI-1 in mediating the MIG-10 outgrowth-promoting activity in the AVM and PVM neurons. In these neurons, the outgrowth-promoting activity of MIG-10 can be oriented by either the UNC-6 or SLT-1 guidance cues [17]. Thus, transgenic expression of MIG-10 does not cause multipolar outgrowth in the wild-type, *unc-6* null, or *slt-1* null backgrounds. However, transgenic expression of MIG-10 does produce multipolar outgrowth in the *unc-6; slt-1* double null background. We found that the *abi-1(tm494)* loss of function mutation significantly suppresses the MIG-10 outgrowth activity in the *unc-6; slt-1* double null mutant background in the AVM and PVM neurons (Figure 4D and Figure S3). These results further support our conclusion that ABI-1 mediates the outgrowth-promoting activity of MIG-10.

### ABI-1 (Abi1) mediates a conserved pathway that promotes formation of lamellipodia downstream of MIG-10

**Figure 5:**
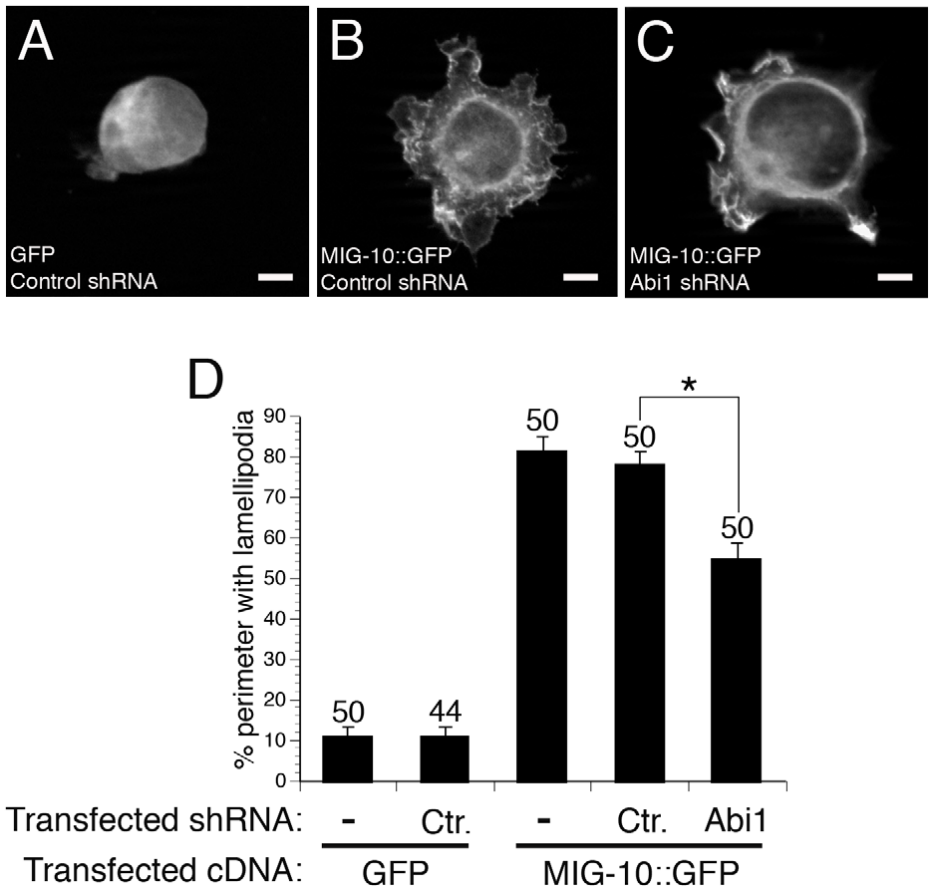
ABI-1 mediates the lamellipodia-forming activity of MIG-10 in cultured HEK293 cells. (A) Example of cell transfected with GFP and control shRNA. (B) Example of cell transfected with MIG-10::GFP and control shRNA. (C) Example of cell transfected with MIG-10::GFP and Abi1 shRNA. (D) Knockdown of Abi1 suppresses the lamellipodia-forming activity of MIG-10. Graph shows the average cell perimeter with lamellipodia. “Ctr.” means cells transfected with scrambled control shRNA. “−” means cells were not transfected with any shRNA. “Abi1” means cells were transfected with the PAV197 shRNA against Abi1. Error bars represent the standard error of the mean. *Bracket indicates statistically significant difference, t-test (p<0.0001). Scale bars are 5 µm.

Overexpression of *C. elegans* MIG-10 in cultured mammalian cells induces the formation of lamellipodia, suggesting that MIG-10 can interact with a conserved pathway to promote the formation of lamellipodia [17]. To determine if ABI-1 might be a part of that conserved pathway, we asked if the mammalian homolog of ABI-1 (Abi1) is required for the lamellipodia-forming activity of MIG-10 in cultured mammalian cells. To address this question, we knocked down expression of mammalian ABI-1 (Abi1) in HEK293 cells expressing MIG-10::GFP (Figure 5). Control cells expressing GFP have a round morphology with only minimal lamellipodia (Figure 5A, 5D). Expression of MIG-10::GFP induces the formation of lamellipodia (Figure 5B, 5D), which is significantly reduced by co-expression of an Abi1 shRNA (Figure 5C, 5D). By contrast, co-expression of a scrambled control shRNA had no effect on lamellipodia formation (Figure 5D). These results indicate that MIG-10 promotes the formation of lamellipodia in mammalian cells by functioning with a conserved pathway that includes mammalian Abi1.

## Discussion

Several actin regulatory proteins have been implicated in the control of growth cone morphology [10]. A few of these have been implicated in the directional response to specific guidance cues. However, little is known about how actin regulatory proteins interact with one another to establish a directional response to guidance cues. Here, we uncover an interaction between the MIG-10 cytoplasmic scaffold and the ABI-1 actin regulatory protein. This interaction could help to explain how actin polymerization is spatially regulated during guidance, since MIG-10 is asymmetrically localized in response to guidance cues [15], [18]. Concurrent work has indicated that the interaction between MIG-10 and ABI-1 can also regulate excretory canal morphogenesis and synapse formation [28], [29], Moreover, recent work has also shown that axon guidance cues and receptors are involved in regulating the WVE-1 complex during embryonic morphogenesis [33]. Together, these observations suggest that the interactions between axon guidance signaling components and the WVE-1 actin regulatory complex may be important in multiple aspects of actin-dependant developmental processes.

Genetic studies of UNC-6 (netrin) signaling in *C. elegans* have revealed a direction-sensing module that includes the UNC-40 receptor, PI 3-Kinase, Rac and MIG-10 [15]–[19]. UNC-6 is secreted from ventrally localized cells and is thought to form a gradient that causes the HSN axon to migrate ventrally. In response to the UNC-6 gradient, UNC-40 becomes asymmetrically localized to the ventral side of the HSN cell. UNC-40 is thought to initiate signaling events that involve activation of Rac and production of PI(3,4)P2 by PI 3-Kinase. MIG-10 binds to both Rac and PI(3,4)P2 and thus becomes asymmetrically localized to the ventral side of the cell. This direction-sensing module is reminiscent of chemotaxis in neutrophils, where Rac and PI 3-Kinase are thought to form a positive feedback loop that transforms directional information from shallow gradients of chemotactic cues into sharply localized directional signal that promotes actin-based motility [34], [35]. Likewise, we propose that UNC-40, Rac, PI 3-Kinase and MIG-10 form a direction-sensing module that can transform a shallow gradient of UNC-6 into a sharply localized outgrowth-promoting activity.

Our current results provide a link that connects MIG-10 to an actin polymerization-promoting complex, thereby explaining how the directional information encoded by asymmetric localization of MIG-10 can be transformed into directed axon outgrowth. We have found that MIG-10 interacts with ABI-1 and WVE-1 to mediate axon guidance. Previous work has indicated that ABI-1 binds to WVE-1 to promote its ability to activate the ARP2/3 complex [21]. The ARP2/3 complex can nucleate actin branches, thereby producing the meshwork of actin that is thought to provide the force that drives motility [36]. Therefore, the interaction between MIG-10 and ABI-1 can explain how MIG-10 is able to spatially direct outgrowth activity. We have been unable to visualize ABI-1::GFP in the HSN neuron. However, studies of Abi1 in mammalian cells indicate that it is located at the leading edge of migrating cells [37]. Since we have found that ABI-1 functions with MIG-10, it is likely that ABI-1 localizes to the leading edge of the HSN neuron. Alternatively, ABI-1 might be localized throughout the cell. However, since MIG-10 is localized to the leading edge, the functional interaction between MIG-10 and ABI-1 would still be confined to the leading edge.

Previous work has implicated ABI-1 and WVE-1 in axon guidance, but the guidance cues were not known [11], [13], [14]. Our current work indicates that ABI-1 and WVE-1 are involved in both the attractive UNC-6 signaling pathway and the repulsive SLT-1 signaling pathway. Despite being involved in both UNC-6 and SLT-1 signaling, the single *abi-1* loss of function mutant does not have any defects in AVM or PVM guidance, suggesting that ABI-1 functions redundantly with other proteins to mediate guidance in these neurons. The lack of AVM and PVM guidance defects in *abi-1(tm494)* mutants might also be due to the fact that this is a hypomorphic allele [32]. In fact, recent work has indicated that RNAi depletion of GEX-3, another member of the WVE-1 complex, can cause mild guidance defects in the AVM [33]. In our study, the role of ABI-1 in axon guidance is revealed in *abi-1; unc-6* or *abi-1; slt-1* double mutants, in which guidance information has been reduced to create a sensitized genetic background. Mutations in several other axon guidance genes (including null alleles) also give only weak or non-existent phenotypes as single mutants, but are enhanced by *unc-6* or *slt-1* null mutations. These mutations include *mig-10(null)*, *age-1(maternally rescued)*, *unc-34(null)*, *ced-10(hypomorphic)*, *unc-115(null)* and *madd-2(null)* 16–18,30,31. Together, these observations suggest that guidance signaling functions with a great deal of redundancy in these neurons.

Our results, when considered with previous findings, suggest that CED-10, MIG-10, ABI-1 and WVE-1 may be organized into a complex or complexes that features redundant physical interactions (see Figure S4). Our previous and current results suggest that CED-10 can interact with MIG-10 and that MIG-10 interacts with ABI-1 [18]. Previous studies have indicated that CED-10 can interact with a subcomplex that contains Sra1 (GEX-2) and Nap1 (GEX-3) [21]. This GEX-2/GEX-3 subcomplex interacts with ABI-1. Thus, ABI-1 could be redundantly bound by both MIG-10 and the GEX-2/GEX-3 subcomplex. This redundant binding could occur within a single complex or could occur within separate complexes (see Figure S4). Discrimination between these two possible configurations will require biochemical and structural studies. We propose that guidance signaling molecules may be organized into networks that include redundant physical interactions. This redundancy could provide a more robust mechanism for the control of the actin polymerization. This model is consistent with the observation that mutations in genes that encode guidance signaling proteins (such as *mig-10*, *age-1*, *unc-34*, *ced-10*, *unc-115* and *madd-2*), generally lead to only weak or nonexistent guidance defects [16]–[18], [30], [31].

In our study, ABI-1 is shown to act in both the UNC-6 and SLT-1 pathways. Several other intracellular outgrowth-promoting proteins have also been implicated in both attractive and repulsive signaling pathways including MIG-10, Rac, Pak, UNC-34 and Abl [16], [17], [38]–[41]. In addition, overexpression of the repulsive UNC-5 receptor can promote neurite outgrowth in cultured cells [42]. Together, these observations suggest that a common set of outgrowth-promoting proteins are involved in both attractive and repulsive responses. A likely explanation for the dual roles of outgrowth-promoting proteins is that they could be oriented by both attractive and repulsive signals, thereby promoting growth towards or away from the source of guidance cue, respectively. Thus, the difference between attraction and repulsion could be in where and how a common set of outgrowth-promoting proteins are localized.

Finally, ABI-1 may link to multiple upstream signaling modules to mediate distinct aspects of axon growth. For instance, recent work has found that the UNC-53 scaffold protein interacts with ABI-1 to promote longitudinal axon growth in *C. elegans* [32]. In *unc-53* loss of function mutants, longitudinal, but not circumferential axon growth is disrupted. Conversely, MIG-10 is thought to mediate circumferential, but not longitudinal axon growth [16], [17]. These observations suggest that the mechanisms that control actin polymerization to drive longitudinal outgrowth are distinct from those that control actin polymerization to drive circumferential guidance. We propose that for the process of circumferential axon guidance, ABI-1 links to the MIG-10 scaffold. By contrast, for the process of longitudinal axon extension, ABI-1 links to the UNC-53 scaffold. Therefore, each of these distinct upstream regulatory processes can both utilize the same actin regulatory complex.

## Materials and Methods

### Strains

AGC1: *slt-1(eh15); abi-1(tm494); cueEx1*, AGC2: *slt-1(eh15); abi-1(tm494) cueEx2*, AGC3: *abi-1(tm494); cueIs3*, AGC4: *cueIs3*, AGC5: *cueIs3; wve-1(ok3308)*/hT2 [*bli4(e937); let-?(q782); qIs48*], AGC6: *slt-1(eh15); abi-1(tm494) cueEx4*, AGC8: *wve-1(ok3308)*/hT2 [*bli4(e937); let-?(q782); qIs48*]; *kyIs262*, AGC9: *mig-10(ct41)*/hT2 [*bli4(e937); let-?(q782); qIs48*]; *kyIs262*, AGC10: *mig-10(ct41)*/hT2 [*bli4(e937); let-?(q782); qIs48*]; *wve-1(ok3308)/*hT2; *kyIs262*, AGC12: *abi-1(tm494)*; *slt-1(eh15)*; *zdIs5*, AGC13: *abi-1(tm494); unc-6(ev400); zdis5*, AGC14: *abi-1(tm494); zdIs5*, AGC15: *cueIs3*; *max-2(nv162)*, AGC16: *wve-1(ne350)*/hT2; *mig-10(ct41)*/hT2, AGC18: *unc-6(ev400)*; *slt-1(eh15)*; *abi-1(tm494)*; *zdIs5*, AGC19: *unc-6(ev400)*; *slt-1(eh15)*; *abi-1(tm494)*; *urEx305*. AGC20: *cueIs7*; *abi-1(tm494); zdIs5*, AGC21: *cueIs7; zdIs5*. The *wve-1(ne350)* allele was a gift from Martha Soto [23], [43]. The *wve-1(ok3308)* allele was obtained from the CGC and out-crossed 3 times. We found that the *ok3308* mutation behaved as a zygotic sterile. The *abi-1(tm494)* hypomorphic allele was provided by Shohei Mitani.

### Transgenes

*cueEx1 and cueEx2* [*mec-4::abi-1; odr-1::dsred]* were created by injecting pAGC2 at 25 ng/ul and *odr-1::dsred* at 50 ng/ul. *urEx305 [mec-4::mig-10a; flp-20::gfp]* was created as described previously [17]. *cueIs3* [*mec-4::mig-10a; flp-20::gfp*] was created by integrating *urEx305* with a gamma radiation source. *cueEx4* [*mec-7::abi-1; odr-1::dsred]* was created by injecting pAGC3 at 25 ng/ul and *odr-1::dsred* at 50 ng/ul. *kyIs262* [*unc-86::myrgfp*] was kindly provided by Cori Bargmann. *zdIs5 [mec-4::gfp]* was obtained from the CGC. *evEx344* [*mec-7::unc-40; unc-129::gfp*] was a gift from Joseph Culotti. *cueIs7* was created by integrating *evEx344*.

### DNA constructs

pAGC2 contains the *mec-4::abi-1* and was created by amplifying the *abi-1* cDNA using the following primers fwd: gcagcagctagccaccatgagtgttaatgatcttcaag and rev: gcagcaggtacctcatactggaactacgtagtttc. The PCR product was cut with NheI and KpnI and ligated into the Nhe1 and Kpn1 sites of pIM207 [17], [44].

pAGC3 contains *mec-7::abi-1* and was created by using Nhe1 and Kpn1 to subclone the *abi-1* cDNA from pAGC2 into pIM211 [17].

pAGC4 contains GST::abi-1-SH3 and was created by using the following primers to amplify DNA encoding the SH3 domain of ABI-1: fwd:gccacaagcgatccagtctctttgatacgagtgct and rev: gccacaaggaattctcatactggaactacgtagtt. The PCR product was cut with BamHI and EcoRI and cloned into the same sites of pGEX-2T.

PAV197 contains an shRNA construct for Abi1 and PAV16 contains a scrambled control shRNA. Both of these constructs were kindly provided by Giorgio Scita [21].

### GST binding assay

GST binding assays were performed as described previously [45]. Briefly, a GST fusion with the SH3 domain of ABI-1 (GST::ABI-1-SH3) was prepared by transforming BL21(DE3) cells with pAGC4. Cell lysates containing MIG-10::GFP were prepared by transfecting HEK293 cells with a plasmid encoding MIG-10::GFP and lysing cells 24 hours later. The GST::ABI-1-SH3 fusion protein was purified and coupled to glutathione-sepharose. Cell lysates were added to the glutathione-GST::ABI-1-SH3 complex and incubated for 2 hours. The glutathione-sepharose-protein complexes were washed three times with 0.1% Triton X-100, boiled in loading buffer and run on and SDS PAGE gel. Bound proteins were detected with an antibody against GFP.

### Cell culture

HEK293 cells were grown in DMEM with 10% FBS. Fugene 6 was used to transfect or co-transfect cells with the appropriate plasmids. For co-transfection experiments 2 µg of DNA encoding the appropriate shRNA was mixed 1 µg of DNA encoding GFP or MIG-10::GFP. Cells were transfected in plastic dishes and allowed to grow for 72 hours after transfection. Next, cells were removed from the plastic dishes and were replated onto glass coverslips coated with polylysine. After 24 hours, the cells were fixed with paraformaldehyde and mounted for observation and analysis.

### Analysis of phenotypes

For analysis of axon guidance phenotypes, animals were mounted on a 5% agarose pad and observed with a 40× objective. For AVM and PVM ventral guidance, an axon was scored as defective if it failed to reach the ventral nerve cord. For HSN ventral guidance, an axon was scored as defective if it traveled laterally for a distance equivalent to 2 cell bodies or more prior to migrating ventrally. For analysis of multipolarity in the ALM, AVM and PVM neurons, a cell was scored as defective if it had a posterior axon that was longer than 2 cell bodies.

## Supporting Information

Figure S1 Loss of *abi-1* function does not enhance PVM ventral axon guidance defects in the *unc-6; slt-1* mutant background. In the PVM axon the *unc-6(ev400)* null allele results in partially penetrant ventral axon guidance defects. The *slt-1(eh15)* null allele results in only rare ventral guidance defects. The double *unc-6; slt-1* guidance defects are highly penetrant, suggesting that UNC-6 and SLT-1 are the predominant guidance cues for PVM ventral axon guidance. These defects are not further enhanced in the *unc-6; slt-1; abi-1* triple mutant.
(TIF)

Figure S2 ABI-1 functions downstram of UNC-40. A *mec-7::unc-40* transgene was used to overexpress UNC-40 in the PVM neuron. Overexpression of UNC-40 caused ventral axon guidance defects in the PVM neuron. These guidance defects were suppressed by an *abi-1(tm494)* loss of function mutation. Error bars represent standard error of the proportion. *Statistically significant difference compared to wild-type animals, z-test for proportions (p<0.05).
(TIF)

Figure S3 Loss of *abi-1* function suppresses MIG-10 outgrowth-promoting activity. The PVM neuron normally has a single process growing out of its cell body. Transgenic expression of MIG-10 in the *unc-6; slt-1* double mutant background produces a multipolar phenotype, where one or more additional processes grow out of the PVM cell body. This outgrowth-promoting activity of MIG-10 is suppressed by the *abi-1(tm494)* loss of function mutation. *Statistically significant difference compared to *unc-6; slt-1* double mutant, z-test for proportions (p<0.0001).
(TIF)

Figure S4 Model for redundant physical interactions between molecules involved in axon guidance. The observations presented in this paper indicate that MIG-10 binds to ABI-1. A previous report has indicated that activated CED-10 binds to MIG-10 [18]. Previous work has also defined the Wave complex, consisting of Sra-1 (GEX-2), Nap1 (GEX-3), Abi1 (ABI-1), and Wave (WVE-1). A subcomplex consisting of Sra-1 (GEX-2) and Nap1 (GEX-3) can bind to activated Rac (CED-10) and also to another subcomplex consisting of Abi1 (ABI-1) and (Wave) WVE-1 [21]. Taken together, these observations suggest that CED-10, MIG-10, ABI-1, WVE-1, GEX-2 and GEX-3 could be organized into a complex that features redundant physical interactions (see upper panel). In this Single Complex Model, CED-10 would be simultaneously bound to both MIG-10 and the GEX-2/GEX-3 subcomplex. Alternatively, these proteins could be organized into two separate complexes, each linking CED-10 to the ABI-1/WVE-1 subcomplex (see lower panel). In this Separate Complex Model, MIG-10 would essentially be doing the function of the GEX-2/GEX-3 subcomplex, which is linking CED-10 and phospholipids to the ABI-1/WVE-1 subcomplex. Discrimination between these two models would require detailed structural and biochemical studies, which have been done for the WVE-1 complex [46], but not for MIG-10. In both models, the ABI-1/WVE-1 subcomplex would be linked to activated CED-10 by two redundant physical interactions, one with MIG-10 and the other with the GEX-2/GEX-3 subcomplex. These redundant physical interactions could provide for a more robust regulation of actin polymerization.
(TIF)
