# Supplementary figures and images for: MIG-10 Functions with ABI-1 to Mediate the UNC-6 and SLT-1 Axon Guidance Signaling Pathways
Source: PLoS Genet. 2012 Nov 29;8(11):e1003054. doi: 10.1371/journal.pgen.1003054 (PMC3510047; doi:10.1371/journal.pgen.1003054)

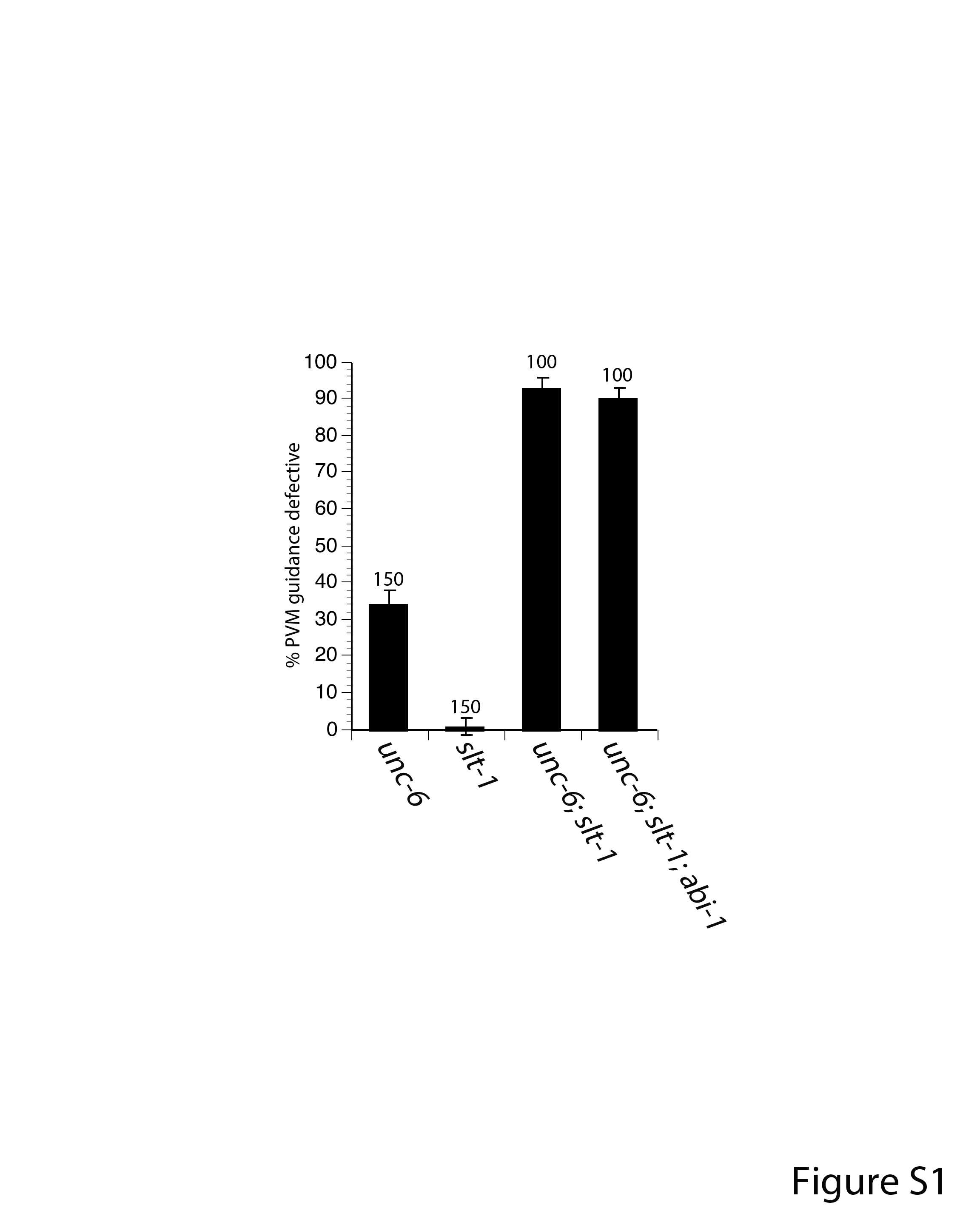

Supplement: Figure S1 — Loss of abi-1 function does not enhance PVM ventral axon guidance defects in the unc-6; slt-1 mutant background. In the PVM axon the unc-6(ev400) null allele results in partially penetrant ventral axon guidance defects. The slt-1(eh15) null allele results in only rare ventral guidance defects. The double unc-6; slt-1 guidance defects are highly penetrant, suggesting that UNC-6 and SLT-1 are the predominant guidance cues for PVM ventral axon guidance. These defects are not further enhanced in the unc-6; slt-1; abi-1 triple mutant. (TIF) [file pgen.1003054.s001.tif]

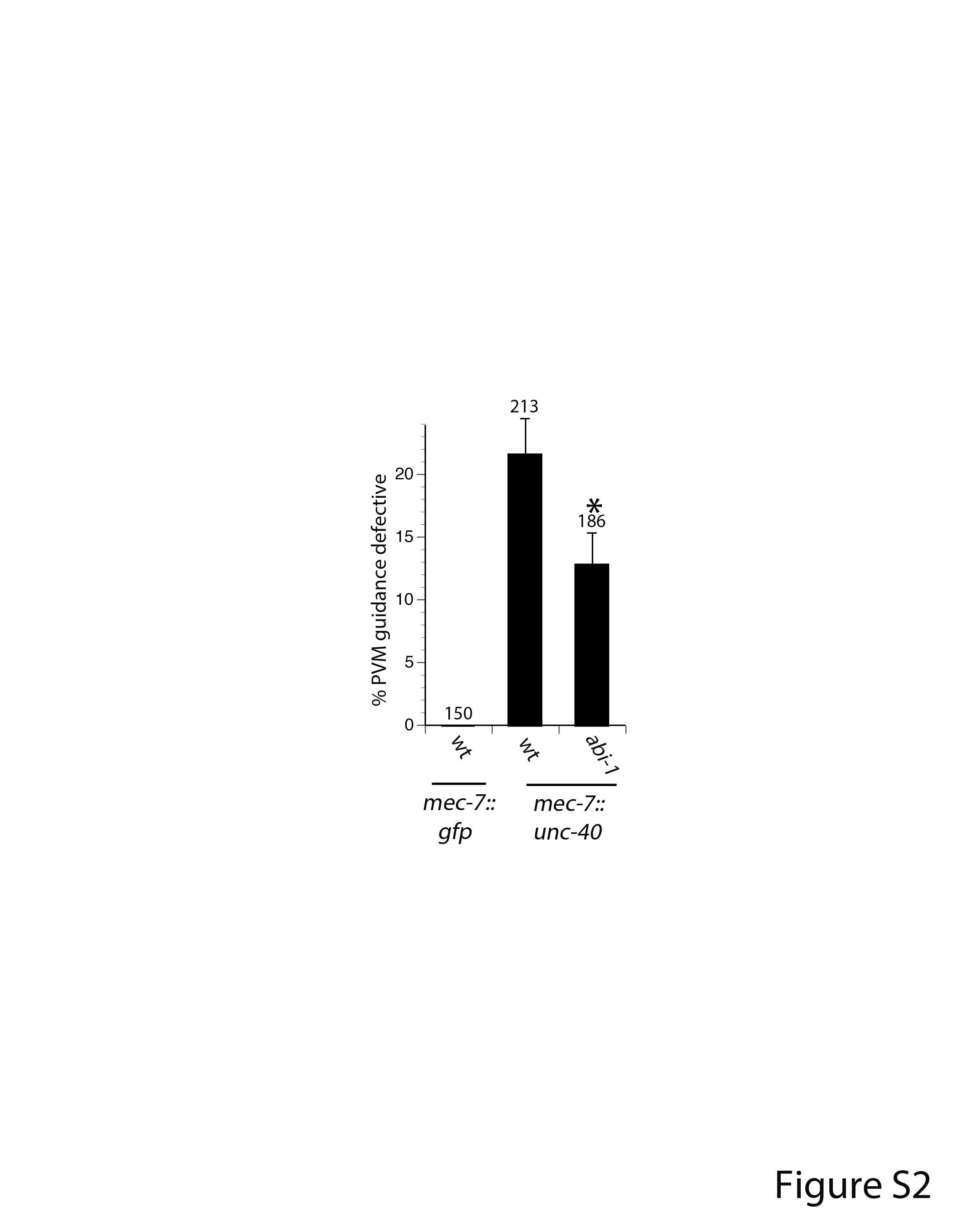

Supplement: Figure S2 — ABI-1 functions downstram of UNC-40. A mec-7::unc-40 transgene was used to overexpress UNC-40 in the PVM neuron. Overexpression of UNC-40 caused ventral axon guidance defects in the PVM neuron. These guidance defects were suppressed by an abi-1(tm494) loss of function mutation. Error bars represent standard error of the proportion. *Statistically significant difference compared to wild-type animals, z-test for proportions (p<0.05). (TIF) [file pgen.1003054.s002.tif]

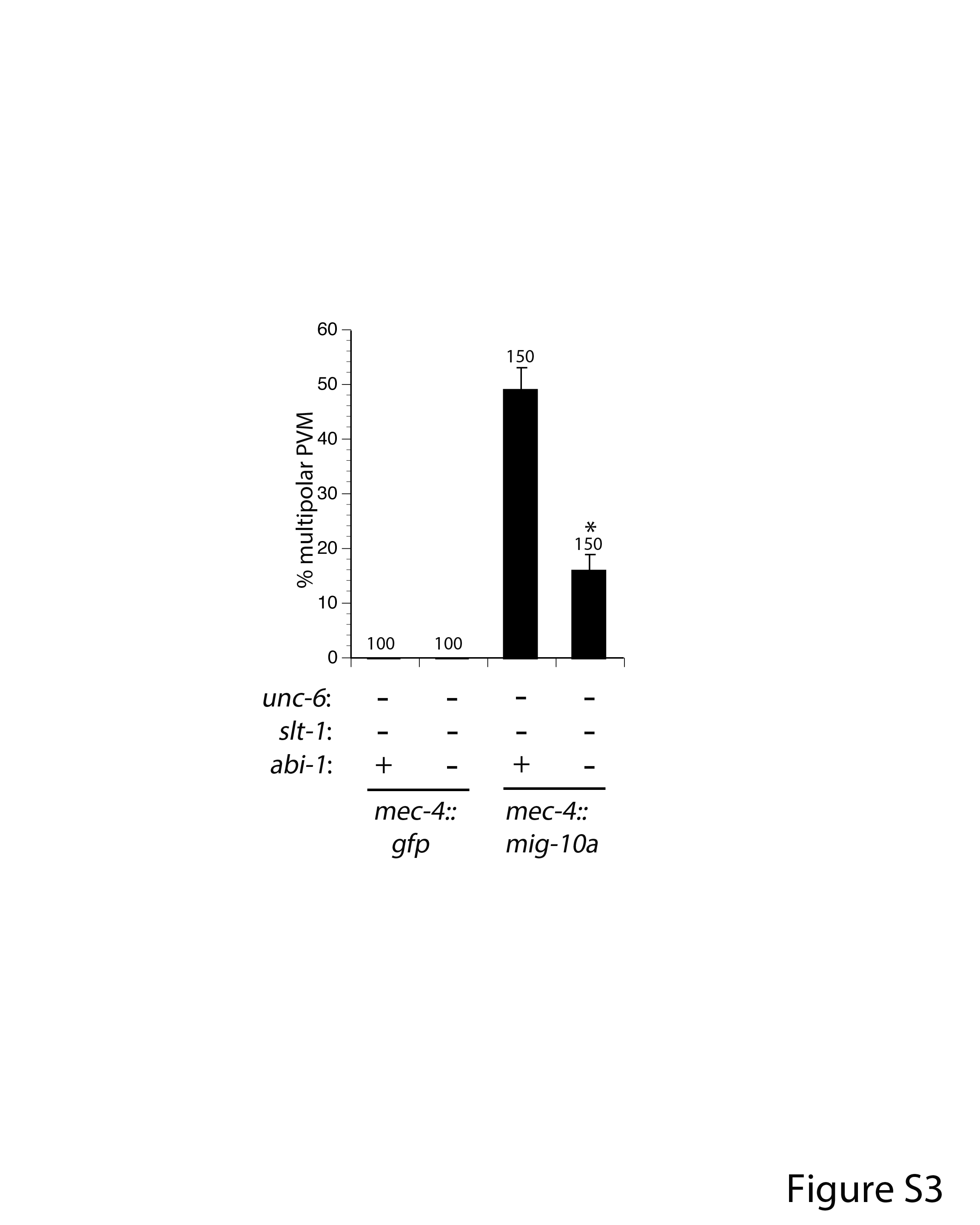

Supplement: Figure S3 — Loss of abi-1 function suppresses MIG-10 outgrowth-promoting activity. The PVM neuron normally has a single process growing out of its cell body. Transgenic expression of MIG-10 in the unc-6; slt-1 double mutant background produces a multipolar phenotype, where one or more additional processes grow out of the PVM cell body. This outgrowth-promoting activity of MIG-10 is suppressed by the abi-1(tm494) loss of function mutation. *Statistically significant difference compared to unc-6; slt-1 double mutant, z-test for proportions (p<0.0001). (TIF) [file pgen.1003054.s003.tif]

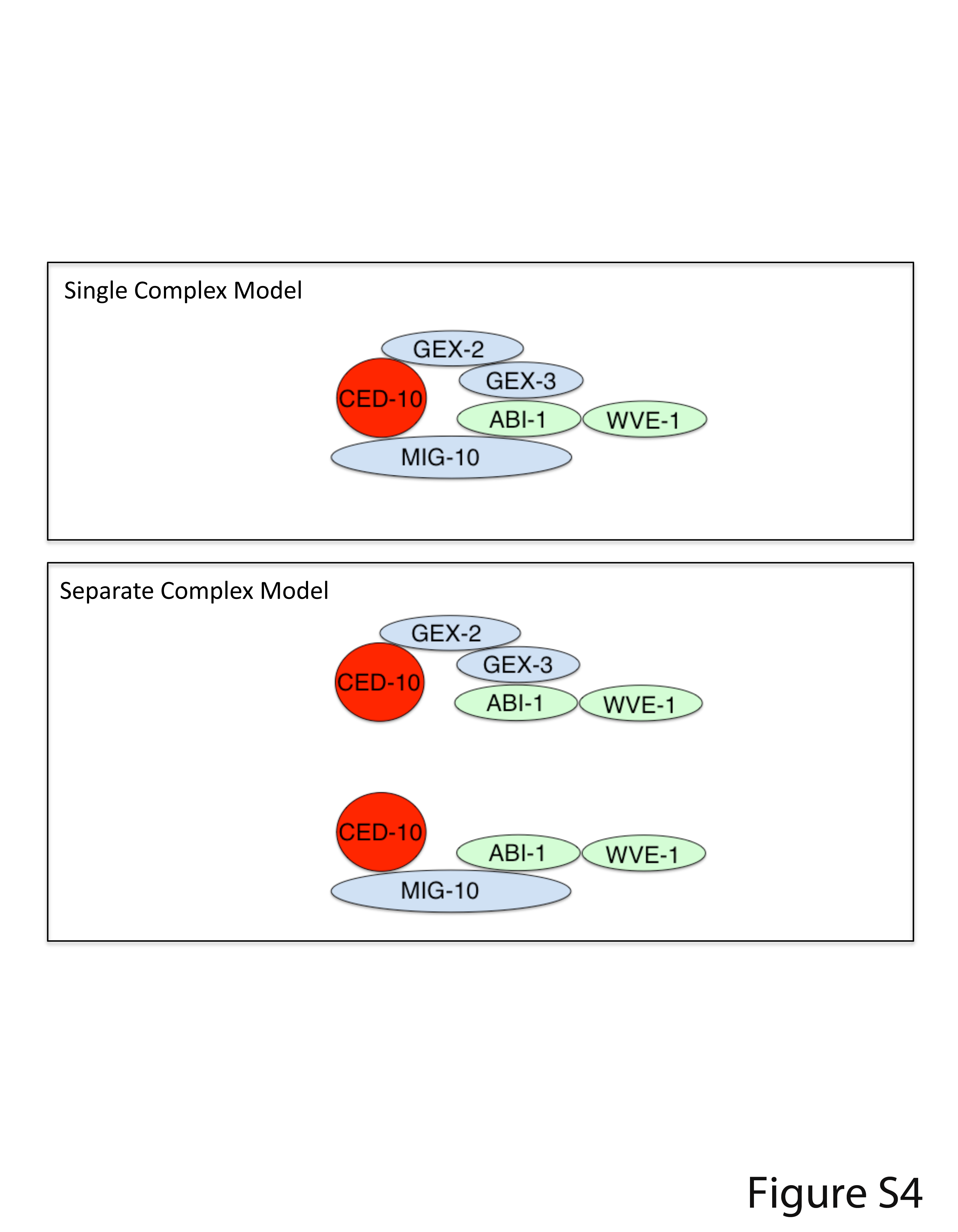

Supplement: Figure S4 — Model for redundant physical interactions between molecules involved in axon guidance. The observations presented in this paper indicate that MIG-10 binds to ABI-1. A previous report has indicated that activated CED-10 binds to MIG-10 [18]. Previous work has also defined the Wave complex, consisting of Sra-1 (GEX-2), Nap1 (GEX-3), Abi1 (ABI-1), and Wave (WVE-1). A subcomplex consisting of Sra-1 (GEX-2) and Nap1 (GEX-3) can bind to activated Rac (CED-10) and also to another subcomplex consisting of Abi1 (ABI-1) and (Wave) WVE-1 [21]. Taken together, these observations suggest that CED-10, MIG-10, ABI-1, WVE-1, GEX-2 and GEX-3 could be organized into a complex that features redundant physical interactions (see upper panel). In this Single Complex Model, CED-10 would be simultaneously bound to both MIG-10 and the GEX-2/GEX-3 subcomplex. Alternatively, these proteins could be organized into two separate complexes, each linking CED-10 to the ABI-1/WVE-1 subcomplex (see lower panel). In this Separate Complex Model, MIG-10 would essentially be doing the function of the GEX-2/GEX-3 subcomplex, which is linking CED-10 and phospholipids to the ABI-1/WVE-1 subcomplex. Discrimination between these two models would require detailed structural and biochemical studies, which have been done for the WVE-1 complex [46], but not for MIG-10. In both models, the ABI-1/WVE-1 subcomplex would be linked to activated CED-10 by two redundant physical interactions, one with MIG-10 and the other with the GEX-2/GEX-3 subcomplex. These redundant physical interactions could provide for a more robust regulation of actin polymerization. (TIF) [file pgen.1003054.s004.tif]
